# Strategies and prospects of effective neural circuits reconstruction after spinal cord injury

**DOI:** 10.1038/s41419-020-2620-z

**Published:** 2020-06-08

**Authors:** Biao Yang, Feng Zhang, Feng Cheng, Liwei Ying, Chenggui Wang, Kesi Shi, Jingkai Wang, Kaishun Xia, Zhe Gong, Xianpeng Huang, Cao Yu, Fangcai Li, Chengzhen Liang, Qixin Chen

**Affiliations:** 1 Department of Orthopedics Surgery, The Second Affiliated Hospital, Zhejiang University School of Medicine, 310009 Hangzhou, Zhejiang China; 2 Zhejiang Key Laboratory of Bone and Joint Precision and Department of Orthopedics Research Institute of Zhejiang University, Hangzhou, Zhejiang 310009 China

**Keywords:** Spinal cord injury, Regeneration, Spinal cord diseases

## Abstract

Due to the disconnection of surviving neural elements after spinal cord injury (SCI), such patients had to suffer irreversible loss of motor or sensory function, and thereafter enormous economic and emotional burdens were brought to society and family. Despite many strategies being dealing with SCI, there is still no effective regenerative therapy. To date, significant progress has been made in studies of SCI repair strategies, including gene regulation of neural regeneration, cell or cell-derived exosomes and growth factors transplantation, repair of biomaterials, and neural signal stimulation. The pathophysiology of SCI is complex and multifaceted, and its mechanisms and processes are incompletely understood. Thus, combinatorial therapies have been demonstrated to be more effective, and lead to better neural circuits reconstruction and functional recovery. Combinations of biomaterials, stem cells, growth factors, drugs, and exosomes have been widely developed. However, simply achieving axon regeneration will not spontaneously lead to meaningful functional recovery. Therefore, the formation and remodeling of functional neural circuits also depend on rehabilitation exercises, such as exercise training, electrical stimulation (ES) and Brain–Computer Interfaces (BCIs). In this review, we summarize the recent progress in biological and engineering strategies for reconstructing neural circuits and promoting functional recovery after SCI, and emphasize current challenges and future directions.

Facts

- A variety of therapeutic strategies, including gene regulation of neural regeneration, cell or cell-derived exosomes and growth factors transplantation, repair of biomaterials, and neural signal stimulation, lead to axonal regeneration and neural circuit reconstruction related to functional recovery.
- The formation and remodeling of functional neural circuits also depend on rehabilitation exercises, such as exercise training, ES and BCIs.
- Combinatorial therapies have been demonstrated to be more effective, and lead to better neural circuits reconstruction and functional recovery.

Open questions

- What are the mechanisms of scar formation after SCI?
- What are the mechanisms that limit the effective formation and regeneration of new neural circuits?
- Is the formation and remodeling of functional neural circuits also depend on rehabilitation exercises, such as exercise training, ES and BCIs?

## Introduction

Spinal cord injury (SCI) is a severely disabling disease that leads to loss of sensation, motor, and autonomic function^1^. Patients with SCI have a higher risk of complications, such as bladder dysfunction, sexual dysfunction, gastrointestinal and respiratory problems, and urinary tract infection^2^, resulting in death in severe cases. The leading cause of SCI in most regions is falls and road injuries^3^, and the other reasons include acts of violence, environmental heat and cold exposure, and sports/recreation activities^4^. About 13% of patients with SCI worldwide suffer from limb dysfunction every year^5^. SCI is a devastating neurodegenerative disorder, which leads to the physical and psychological problems of patients and brings an enormous economic burden on patients, their families and the social medical system^6^.

**Fig. 1:**
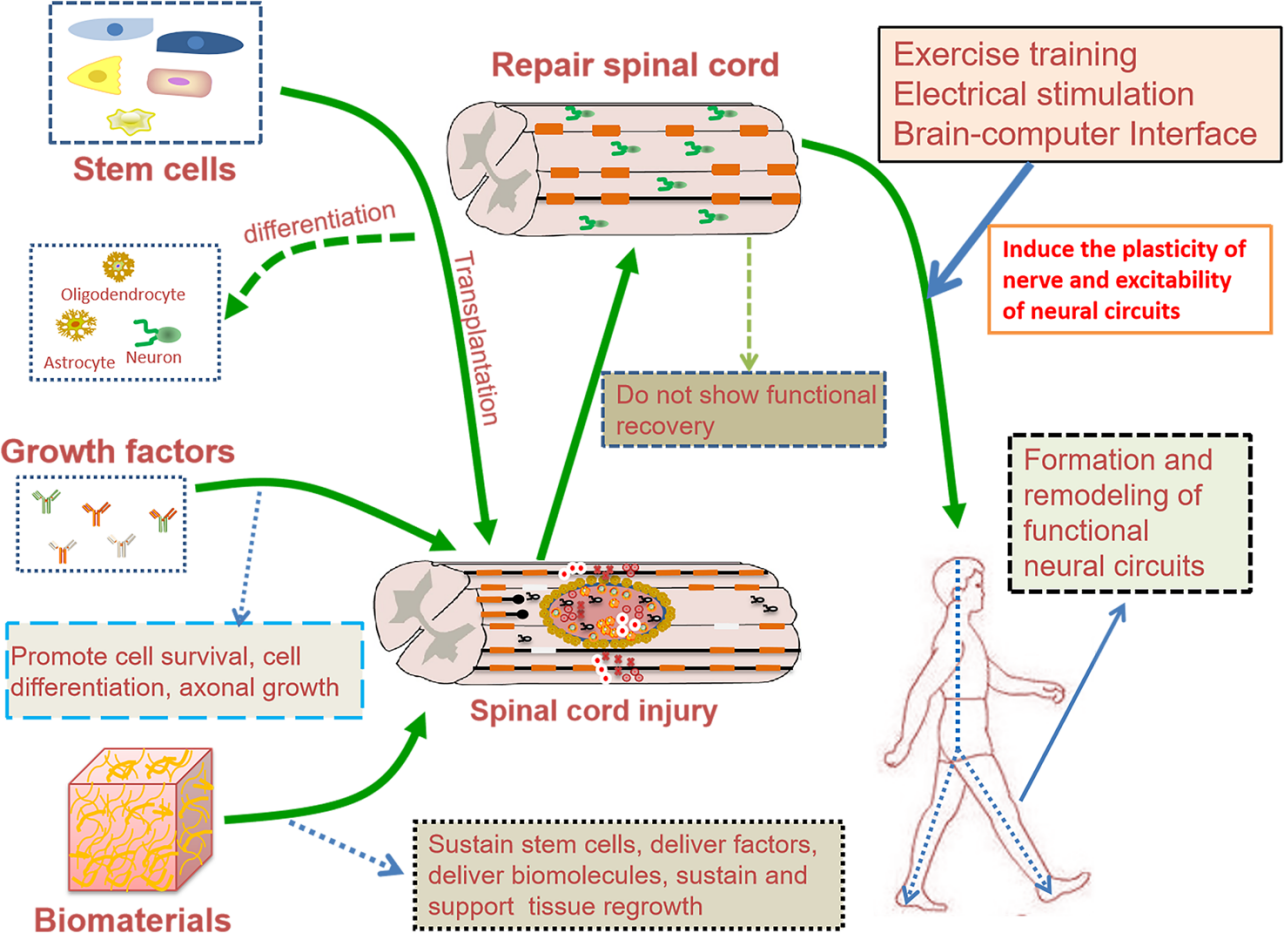
The figure of effective neural circuits reconstruction after SCI. Combinatorial therapies have been demonstrated to be more effective, and lead to better functional recovery. However, simply achieving axon regeneration will not spontaneously lead to meaningful functional recovery. Therefore, the formation and remodeling of functional neural circuits also depend on rehabilitation exercises, such as exercise training, ES and BCIs.

The permanent disability after SCI is related to the failure of axon regeneration and neural circuit reconstruction^1^. To date, there are no effective treatments that can completely regenerate axons after SCI. Over the past decade, significant progress has been made not only in traditional research fields, such as inflammation, scar formation, cell transplantation, axon regeneration, and biomaterial repair, but also in determining the mechanisms of spinal cord automation, spontaneous circuit reorganization and functional recovery after SCI^7,8^. Because of the complexity issues of pathological processes that occur following SCI^9^, combinatorial strategies that solve the problems caused by different aspects are expected to be more effective, and lead to better functional recovery^10^. Combinations of biomaterials, stem cells, growth factors, drugs, and exosomes have been widely developed (Fig. 1). However, functional recovery depends on strengthening neuroplasticity to promote the growth of injured and spared axons, to increase the strength of the remaining connections and to promote the formation of new spontaneous circuits^10^. Therefore, the formation and remodeling of functional neural circuits also depend on rehabilitation exercises, such as exercise training, ES and BCIs (Fig. 1). In this review, we summarize the recent progress in biological and engineering strategies for reconstructing neural circuits and promoting functional recovery after SCI, and emphasize current challenges and future directions.

## Pathophysiology

**Fig. 2:**
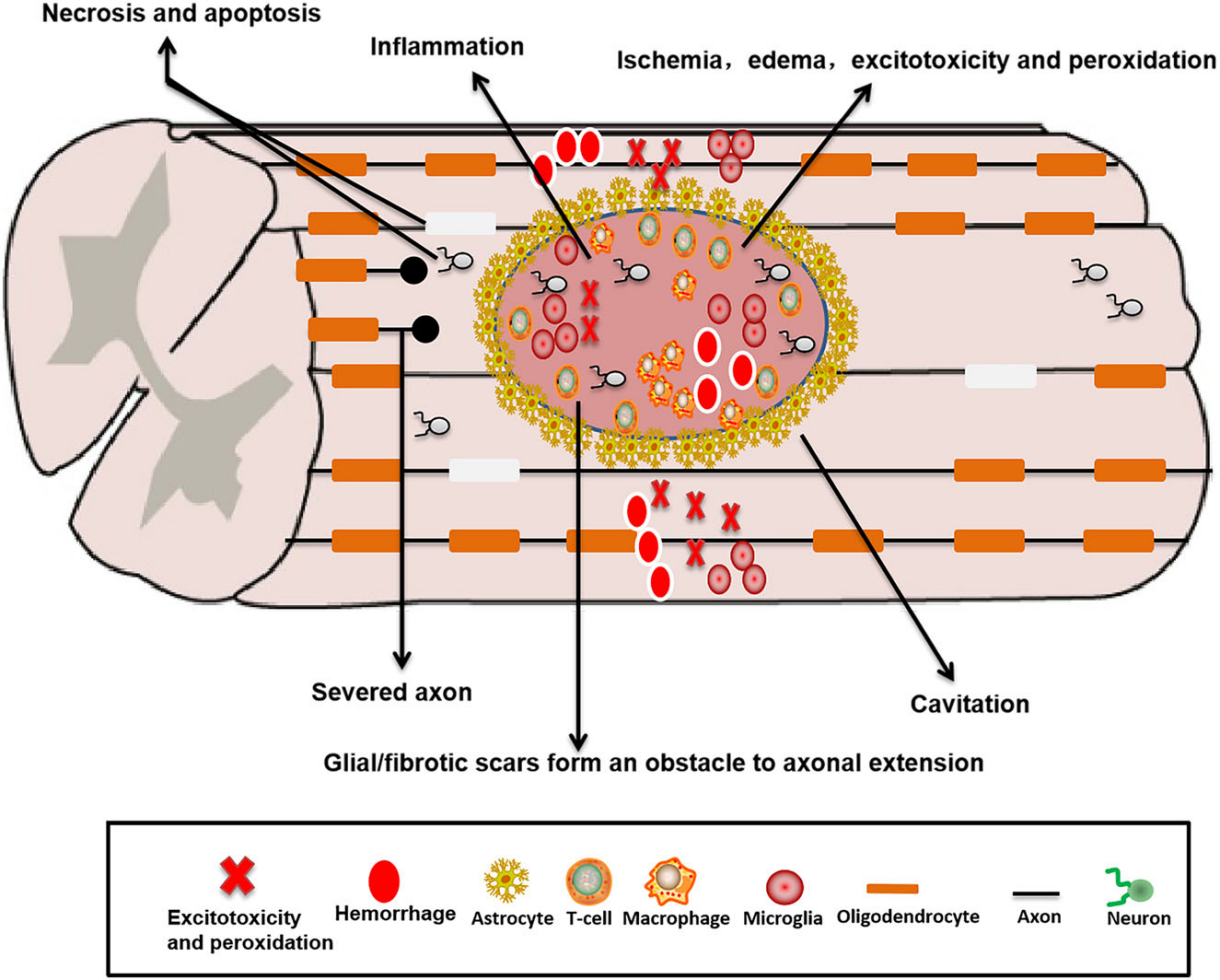
Pathophysiological mechanism of SCI. During SCI, the primary injury leads to the production of free radicals and a chronic state of causing ischemia and hypoxia, resulting in glutamate excitotoxicity, lipid peroxidation, calcium influx, edema and cellular damage. Finally, inflammation and immune response affect the integrity of adjacent tissues. Secondary injury leads to demyelination of the axons, glial cell proliferation, the loss of damaged cells and the disconnection of living neurons, culminating in formation of a microenvironment that is not conducive to nerve regeneration.

SCI is mainly caused by the mechanism of primary and secondary injury (Fig. 2). The primary injury is irreversible, which is related to the initial traumatic damage such as compression, stretch, laceration and hemorrhage, thus triggering a complex cascade of acute and chronic degenerative events that further disrupt neuronal function^11^. During SCI, the primary injury leads to the production of free radicals^12^ and a chronic state of causing ischemia and hypoxia^13^, resulting in glutamate excitotoxicity, lipid peroxidation, calcium influx, edema, and cellular damage^14^. Finally, inflammation and immune response affect the integrity of adjacent tissues^14^. Secondary injury leads to demyelination of the axons, glial cell proliferation, the loss of damaged cells and the disconnection of living neurons, culminating in formation of a microenvironment that is not conducive to nerve regeneration^15^. In addition, in the chronic stage, glial/fibrotic scars that inhibit cellular infiltration^16^ can form an obstacle to axonal regeneration and extension^17^. Astrocytic scars are widely regarded as the main cause that transected axons fail to regrow after SCI^18^, but when pericyte-derived fibrotic scars are reduced at the lesion site, axonal regeneration and functional recovery will be promoted^19^. Extracellular matrix (ECM) proteins deposited within and around glial/fibrous scars limit axon growth, such as chondroitin sulfate proteoglycans (CSPGs), fibronectin, laminin, and collagen. Among them, CSPGs play a major role in inhibiting axon regeneration^20^, including aggrecan, neurogen and neural/glial antigen^21^. Furthermore, myelin-associated inhibitors that strongly inhibit axon and myelin regeneration, including myelin-associated glycoprotein (MAG), Nogo, netrin, ephrins, and oligodendrocyte-myelin glycoprotein (OMgp), can lead to growth cone collapse after SCI^22^. Poor axon growth ability and inhibitory factors that hinder axon regeneration in the scar^23^ lead to the failure of regeneration and repair of spinal cord and reconstruction of neural circuits. Therefore, the therapeutic focus is on how to promote axon regeneration, myelinate axons and reconstruct neural circuits.

## Stem cells transplantation for SCI

**Fig. 3:**
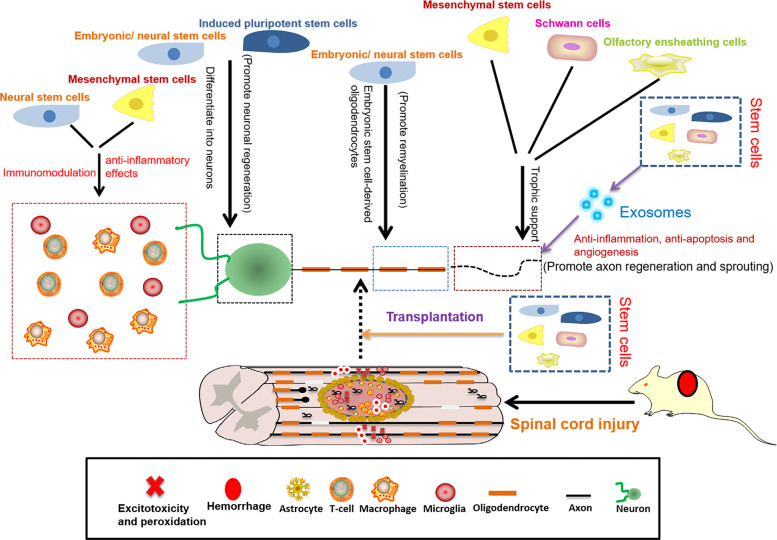
Scheme summarizing the role of each type of stem cells in repair of SCI. Stem cells play a neuroprotective role by differentiating into specialized cell types to replace damaged cells and secreting factors to promote the survival and activity of these cells. In addition, the mechanisms of stem cells that promote repair and function improvement include immunomodulation, anti-inflammatory effect, inhibition of scar formation, axon and myelin regeneration, and prevention of vascular loss or promotion of angiogenesis.

Stem cells play a neuroprotective role by differentiating into specialized cell types to replace damaged cells and secreting factors to promote the survival and activity of these cells^24,25^ (Fig. 3). In addition, the mechanisms of stem cells that promote repair and function improvement include immunomodulation, anti-inflammatory effect, inhibition of scar formation, axon and myelin regeneration, and prevention of vascular loss or promotion of angiogenesis^24–26^ (Fig. 3).

### Neural stem/progenitor cells

Neural stem/progenitor cells (NSCs/NPCs) can differentiate into neurons for the repair of nerve tissue damage caused by SCI, and into oligodendrocytes to promote axon regeneration and myelination^25,27^. NSCs/NPCs also can regulate astrocytes to protect tissue integrity^28^ and improve the microenvironment of the injury site by secreting factors^15,24^. For instance, a study showed that transplantation of the spinal cord-derived NSCs into the injury site produced massive amounts of excitatory neurons and axons that extend a long distance, leading to the robust regeneration of corticospinal cord^29^. Another study showed that nine months after transplantation of human spinal cord-derived NPCs into the lesion site of the spinal cord, the grafts successfully differentiated into neurons and glial cells that reconstruct the neural tissue and local microenvironment, and the regeneration axons of the host and the grafts successfully were interconnected^30^. However, due to the tendency that NSCs differentiate into astrocytes^31^, some measures, including ES^32^, gene modification^33^, and the application of nanomaterials^34^, should be taken. For instance, Wnt4-modified NSCs could activate MAPK/JNK and β-catenin pathway and inhibit the activation of Notch signaling to significantly promote the differentiation of NSCs into neurons, thus effectively repairing the damaged spinal cord and promoting the recovery of motor function^33^.

### Mesenchymal stem cells

Mesenchymal stem cells (MSCs) can be obtained from different types of tissues, including adipose tissue, placenta, umbilical cord, amniotic membrane and bone marrow^25,31^, which can be expanded efficiently in vitro^25^. Moreover, MSCs have the advantages of high biosafety, low immunogenicity, wide biological effects and immunomodulatory characteristics^35^. MSCs can differentiate into neurons and glial cells after transplantation into the injury site^35^ to reduce the area of glial scar and the size of the cavity, leading to the regeneration of nerve tissue, axon and myelin sheath^36^. The use of MSCs for SCI has a wide range of biological mechanisms, including regulating immune response^37^, promoting angiogenesis^38^, anti-apoptotic and anti-inflammatory effects^39,40^. However, the most important mechanism is the paracrine effect of MSCs, which can release a variety of nutritional factors that protect the injury spinal cord tissue and promote nerve regeneration^24^. In a separate study, the combination of MSCs and three-dimensional (3D) gelatin sponge could form a perineurium-like sheath that enwrapped the host myelin sheaths and axons, and form a physical and chemical barrier to protect the nerve fibers from oxidative damage by secreting nutrients, extracellular matrix and antioxidant superoxide dismutase-3 (SOD-3)^41^. Some strategies such as gene modification, hypoxia preconditioning and improvement of the local microenvironment of the host can promote the survival and differentiation of transplanted MSCs^42,43^. For instance, a recent study found that retinoic acid (RA), sonic hedgehog (SHH), and neurotrophic factors could efficiently induce human adipose-derived stem cells (hADSCs) into electrophysiologically active motoneuron-like cells (hADSC-MNs) in vitro^44^. After hADSC-MNs were transplanted into the injury site, hADSC-MNs could survive well and improve the microenvironment by immunosuppression, thus promoting the reconstruction of the spinal circuit.

### Embryonic stem cells

Embryonic stem cells (ESCs) can differentiate into three types of germ cells due to their great developmental and unlimited self-renewal ability^45^. Many transcription factors, including Nanog, Sox2 and OCT3/4, have been identified in human ESCs^45^, which are used to induce fibroblasts and other somatic cells into pluripotent stem cells (iPSCs)^46^. ESCs can differentiate into neurons and glial cells to repair damaged cells, and secrete active factors to regulate the immune response and provide nutrition support, ultimately leading to the regeneration of nerve tissue and axons, remyelination of axons, and the reconstruction of injury cortical circuits^35,47–49^. For instance, a study conducted by Manley et al.^50^ showed that oligodendrocyte progenitor cells (OPCs) derived from human ESCs could reduce the cavity of the injury site, increase the sparse myelinated axons, and significantly improve the motor function. Another recent study reported that human ESCs derived-NSCs significantly promoted recovery of motor function by reducing the size of the cavity and the area of glial scar^51^.

### Induced pluripotent stem cells

Because previous stem cell sources such as ESCs and MSCs are still plagued by the immune, ethical and clinical concern, iPSCs are an alternative with significant advantages^52^. iPSCs were initially obtained by viral transduction of transcription factors in differentiated somatic cells, such as tumour suppressor Krüppel-like factor 4 [KLF4], OCT3/4, Sox2, and proto-oncogene c-MYC^53^. IPSCs also can be obtained from somatic cells after genetic reprogramming by the combination of other transcription factors^54^ and reprogramming molecules^55,56^ that promote the expression of core transcription factors such as Oct4, Sox2 and Nanog^57^. iPSCs can be differentiated into neurons, astrocytes and oligodendrocytes to replace damaged cells^58^, regulate the microenvironment of the spinal cord by secreting growth factors^59^, and promote the regeneration of axons and myelin sheath, thus reconstructing the neural circuit that promotes functional recovery^60^. For instance, a recent study found that three months after transplantation of iPSCs-derived NSCs into the injury spinal cord, iPSCs-derived NSCs survived and successfully differentiated into neurons, which extended a large number of axons from the injury site and formed synapses with host neurons, leading to the reconstruction of the neural network^61^. Another study conducted by Fan and his colleagues showed that iPSCs-derived NSCs loaded in 3D gelatin methacrylate (GelMA) hydrogel reduced inflammation by reducing activated macrophages/microglia, inhibited the formation of glial scar, and reduced the area of the cavity, thus promoting axonal regeneration after SCI^62^.

### Olfactory ensheathing cells

Olfactory ensheathing cells (OECs) promote axon regeneration and remyelination by inhibiting inflammation of the spinal cord, reducing the glial scar and cavity^63^, and promoting angiogenesis, contributing to the reconstruction of the injury neural network^64^. Moreover, OECs have shown higher levels of growth factors secretion, which can promote the survival and activity of neurons and their axons extension, thus improving tissue repair and functional recovery^65,66^. For example, a recent study found that intravenous OECs transplantation into the injury site significantly decreased local inflammation, thus promoting axon and myelin sheath regeneration related to function recovery^67^. Combinatorial therapies can be used to improve the regeneration efficiency of OECs in the SCI model. For instance, another recent study reported that combining adipose tissue-derived stromal cells (ASCs) with strong paracrine effect with OECs that can induce axonal growth could significantly improve the motor function by reducing the inflammatory response and astrocyte proliferation^68^.

### Schwann cells

Schwann cells (SCs) are the main glial cells in the peripheral nervous system (PNS), which are widely used in the repair of SCI because of their role in neuroprotection, promoting axon regeneration and myelination^25,69^. SCs play a neuroprotective role by inhibiting the activity of pro-inflammatory microglia and macrophages to regulate the local microenvironment^70^. Besides, SCs can secrete a variety of active factors to reduce nerve cell death and the cavity formation^25^. However, combinatorial strategies are often used to improve the effect of SCs transplantation. For example, a study showed that ECM-derived matrices such as Matrigel were used to carry SCs, which improved the survival of SCs in the transplanted site of SCI, and significantly promoted the growth of axons to improve the motor function^71^.

### Exosomes derived from stem cells

The mechanisms of stem cell-based therapy are mediated by exosomes released by stem cells, and microRNA in these exosomes is the main factor to play the therapeutic effect^72^. Exosomes can transmit biological signals between cells and participate in a variety of physiological and pathological processes^73^. Exosomes derived from stem cells are expected to be a new strategy for the treatment of SCI. Exosomes have the functions of neuroprotection, angiogenesis, immune regulation, anti-inflammatory, anti-apoptosis, and axon regeneration, which can promote the repair and regeneration of nerve tissue^74,75^. For example, a study showed that the human umbilical cord mesenchymal stem cells (hucMSC) derived exosomes could effectively induce macrophages derived from bone marrow to polarize from M1 phenotype to M2 phenotype, and reduce inflammatory factors such as IL-6, IFN-γ and TNF-α, thus promoting functional recovery after SCI^76^. Another study found that NSCs-derived exosomes reduced the extent of SCI, repaired the injury site, and promoted the functional recovery by reducing the neuroinflammatory response, microglia activation and neuronal apoptosis^77^. Combined with the advantages of exosomes, exosomes for molecular targeted drug delivery are expected to become the next generation of intelligent engineering biomaterials for precision medicine^78^. For instance, as shown in the study by Kim et al., iron oxide nanoparticle (IONP)-incorporated exosome-mimetic nanovesicles from IONP-treated human mesenchymal stem cells (hMSCs) could target the injury spinal cord under magnetic guidance^79^. Compared with the exosomes from hMSCs alone, it significantly reduced inflammation, inhibited apoptosis and promoted vascular regeneration, leading to significant improvement in functional recovery after SCI. In another study, MSCs-derived exosomes loaded with phosphatase and tensin homolog siRNA significantly reduced the proliferation of astrocytes and microglia, promoted the formation of new blood vessels and axons regeneration, thus promoting the functional recovery after SCI^80^.

## Biomaterials

Biomaterials can improve the local microenvironment and promote the survival of nerve cells by reducing the inflammatory response, improving cell retention, inhibiting cell necrosis or apoptosis, and reducing the formation of the glial scar, thus reconnecting the injury spinal cord tissue, reconstructing the nerve conduction circuit, and ultimately promoting the motor recovery after SCI^81^. As a method of regenerative medicine, biomaterials containing cells, drugs and bioactive molecules can repair or replace the function and anatomical structure of the damaged tissue^82^. However, the main neural mechanism of functional biomaterials that promote motor function recovery depends on the formation of neuronal bridging, rather than the long-distance regeneration of the descending motor axon and ascending sensory axon^83^. Although various forms of biomaterials can be used, hydrogels, nanomaterials and 3D printing materials represent an attractive form for spinal cord repair.

The hydrogel has the characteristics of the porous structure, high water content and sol-gel transformation under certain conditions, which is widely used in tissue engineering, cell therapy and drug delivery^84^. Therefore, the hydrogel is currently an ideal carrier because it can mimic ECM to provide suitable microenvironment and support for stem cells^85^. Moreover, the hydrogel can provide the proper 3D networks for axonal growth, and promote axonal regeneration, synaptic reconnection and reconstruction of neural circuits by carrying various drugs and growth factors^8^. For instance, a recent report by Caron et al. described the use of 3D biomimetic agarose/carbomer hydrogel and hMSCs that optimize the density, vitality and paracrine transport could significantly increase M2 macrophage population to improve the microenvironment of the injury site^86^. Papa et al.^87^ further found that human chemokine (C–C motif) ligand 2 (hCCL2) secreted by hMSCs could promote macrophage recruitment and transformation to M2 phenotype to play a neuroprotective role, thus preventing motor neuron degeneration to improve the motor function after SCI. The responsive hydrogel can respond to stimuli such as ions, pH, temperature, enzyme and electromagnetic fields, resulting in changes in its physical and chemical properties to better mimic the dynamic natural 3D cell microenvironment^88^. For example, a study found that composite hydrogels consisting of alginate, chitosan and genipin were high sensitivity to Ca_2_^+^ concentration, which could alleviate the secondary injury caused by the overload of Ca_2_^+^ and control the astrocyte behavior, leading to the recovery of motor function^89^. In addition, the spinal cord can conduct and transmit bioelectric signal to play a vital role in promoting axon growth, nerve regeneration^90^ and nerve circuits remodeling^91^. For instance, a study showed that conductive hydrogels could inhibit astrocyte development and promote differentiation of NSCs into neurons, thus improving the recovery of motor function after SCI^92^.

3D printing technology allows the manufacture of precise anatomical and personalized scaffolds to stimulate and guide axonal regeneration and elongation. The injury host axons regenerate into 3D printing scaffolds, while implanted NPCs extend axons out of the scaffolds and form synaptic connections with each other to restore synaptic transmission, thus significantly improving the host motor function^93^. To improve biocompatibility or for a special purpose, 3D printing scaffolds can be modified, biochemical functionalized and mechanically tuned to promote stem cell proliferation, adhesion, and differentiation^94^. In a separate study, 3D printing poly-lactic-co-glycolic acid (PLGA) scaffolds provided a favorable 3D environment for the regeneration of nerve cells and neurogliocytes^95^. Another recent study conducted by Sun et al. showed that 3D printing collagen/chitosan scaffolds could promote neural regeneration, reduce scar formation, and partially reconstruct the permissive microenvironment of axon regeneration, ultimately promoting functional recovery^96^. Joung and his colleagues put NPCs and OPCs from iPSCs in the precise position of 3D printing scaffolds to construct the bionic spinal cord, which is helpful to reconstruct the functional axon connection of the injury site^97^.

Due to their unique structures and properties, nanomaterials can be used in the diagnosis and treatment of neurological diseases, and be designed as drug delivery carriers that cross the blood–brain barrier (BBB) and deliver specific molecules to target cells, which have better safety and efficacy than traditional simple drug treatment^98^. Besides, nanomaterials can promote the extravasation and system clearance of the BBB and maintain the high activity of neural cells after entering cells, and surface modification of nanomaterials can also enhance biocompatibility and selective targeting via cell surface receptor interaction^99^. For instance, a study conducted by Papa et al. showed that poly-ε-caprolactone (PCL) nanoparticles (NPs) loaded with minocycline could selectively target and regulate the activated pro-inflammatory microglia to reduce the secondary injury and improve the microenvironment of the injury site, thus promoting axon regeneration^100^. Another study conducted by the same research group showed that a nano-structured gel loaded with rolipram targeting A1 astrocytes could reduce nitric oxide synthase (iNOS) and Lipocalin 2 (Lcn2) to reduce inflammation caused by activated astrocytes, thus protecting nerve cells and promoting the recovery of motor function after SCI^101^. Our group constructed targeting scar tissue and esterase-responsive nano-micelles. After being activated, the micelles were endocytosed and degraded by microglia, resulting in a decrease in accumulation of reactive oxygen species (ROS) and the transformation of M1 to M2 microglia population, thus protecting nerve cells and promoting the recovery of motor function^102^.

## Growth factors

Growth factors are a family of proteins regulating biological development and neural function, which can regulate the survival of neurons, promote the release of neurotransmitters, promote the recovery of synaptic function, and induce the growth and remodeling of axons^103^. However, various types of growth factors have different functions in repairing SCI. For example, neurotrophin-3 (NT-3), neurotrophin-4 (NT-4) and neurotrophin-5 (NT-5) have the function of protecting damaged neurons and promoting the growth and differentiation of neurons^24^. Nerve growth factor (NGF) can support the survival of neurons and promote the growth, differentiation and synapse formation of neurons, thus promoting nerve regeneration, neural network remodeling and motor function recovery^104^. Brain-derived neurotrophic factor (BDNF) has neuroprotective effects on 5-serotonin, dopaminergic, cholinergic and GABA neurons by promoting the growth of neurons, the regeneration and sprouting of axons, and the formation of remyelination of axons^103^. Glial cell-derived neurotrophic factor (GDNF) can reduce inflammation and lesion volume, improve pain, and promote axon regeneration, which also has a positive regulatory effect on astrocyte proliferation^105^. Fibroblast growth factors (FGFs), including FGF1, FGF2, FGF4 and FGF10, can alleviate the secondary injury such as inflammation and astrocyte activation, stimulate axon regeneration and angiogenesis, and protect damaged neurons^106^. Insulin-like growth factor 1 (IGF-1) plays an antioxidant and pro-survival role to promote the differentiation and survival of oligodendrocytes and the growth and survival of axons, and increase the formation of regenerated axonal myelin sheath^107^. Ciliary neurotrophic factor (CNTF) can promote the survival of damaged neurons and oligodendrocytes and the long-distance regeneration of axons^108^. Given the complexity of the pathological process following SCI, it is necessary to use multiple growth factors according to different pathological mechanisms and the effects of growth factors. For example, a study showed that osteopontin, IGF-1 and CNTF could reactivate the growth ability of spinal neurons, FGF2 and epidermal growth factor (EGF) could induce growth support, and GDNF could chemically attract axon growth. A combination of above growth factors whose cooperative effects were required to stimulate the regeneration of robust intrinsic axons, thus enhancing recovery after SCI^109^. Due to the short half-life of growth factors, they are often delivered in combination with biomaterials. A study found that chitosan biomaterials were used for sustained release of NT-3 into the injured spinal cords, where this combinatorial therapy could promote the activation of endogenous NSCs to differentiate into neurons, thus reconnecting the damaged axons to form functional neural networks, leading to the recovery of function^110^. We previously reported that loaded FGF4 heparin-laponite hydrogels could significantly reduce inflammation, inhibit astrocyte proliferation and scar formation, and promote axonal regeneration and remyelination of axons by increasing stable microtubules and activating mitochondria release^111^.

## Rehabilitation

**Fig. 4:**
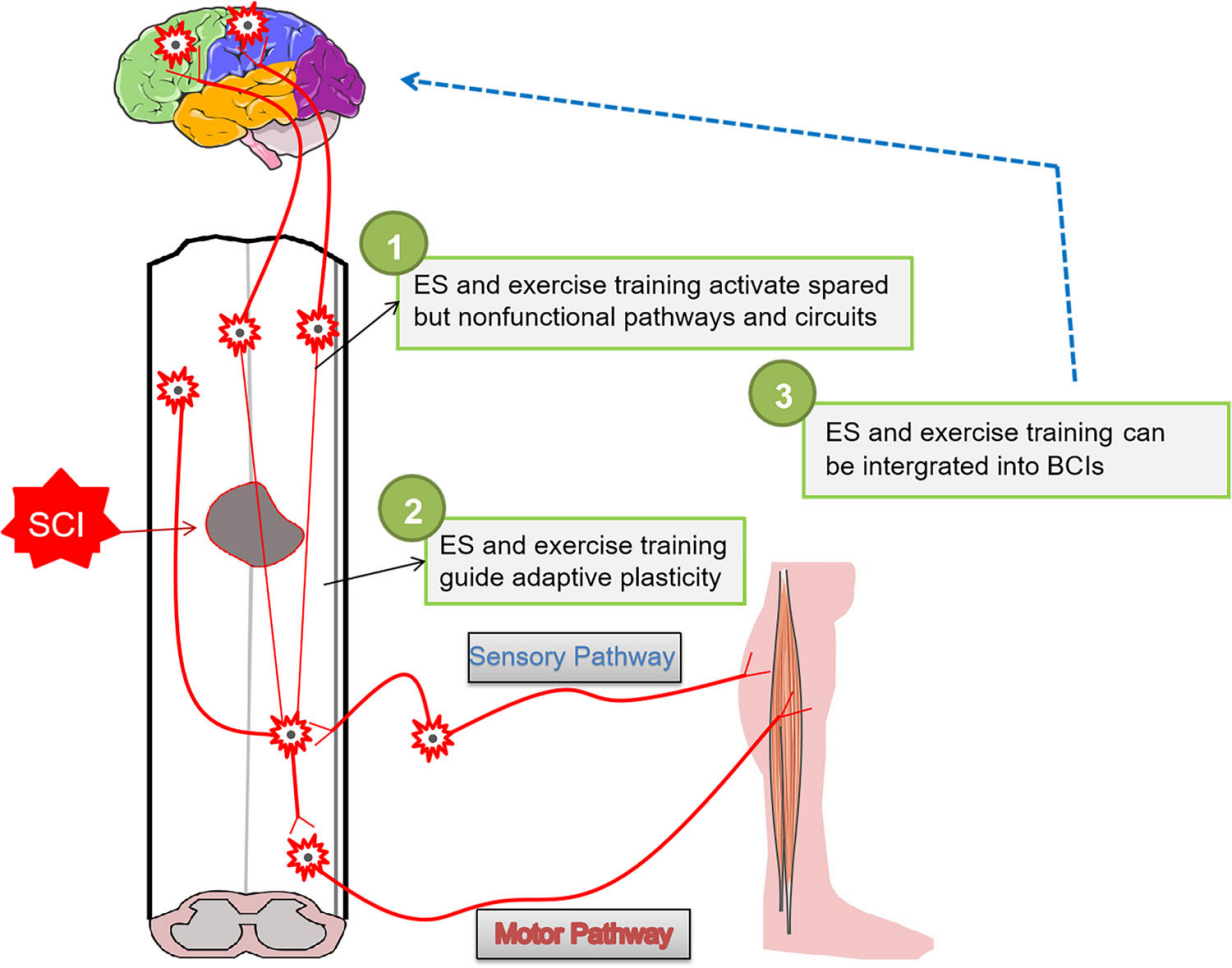
Rehabilitation strategies for the long-term recovery of effective neural circuits. Exercise training, ES and BCIs can regulate the excitability of spinal circuit through the central mechanism, leading to muscle contraction, which can not only restore muscle strength and quality, but also induce the plasticity of nerve and excitability of neural circuit, thus promoting the recovery of motor function.

After SCI, the ascending sensation and descending input of the spinal cord is interrupted, and the axon of corticospinal cord retracts so that it cannot regenerate, resulting in permanent loss of function^112^. However, simply achieving axon regeneration will not spontaneously lead to meaningful functional recovery. Therefore, it is widely recognized that the formation and remodeling of functional neural circuits also depends on strengthening neuroplasticity^113^, such as exercise training, ES and BCIs (Fig. 4). Exercise training can improve the function of spinal motor neurons and the remodeling function of the cerebral cortex, thus increasing neuronal activity to strengthen the reorganization of neural pathways^114^. ES can regulate the excitability of the spinal circuit^115^, restore muscle strength and quality, and induce the plasticity of nerve, thus promoting the recovery of function^116^. BCIs are an emerging technology that translates brain activity into motor actions^117^, which enable direct communication between the brain and electronic devices, thus recording and modulating neural activity to restore lost function^118^.

### Exercise training

Exercise training can reduce apoptosis, increase growth factor levels such as IGF, BDNF, and NGF, decrease inflammation, and promote neurogenesis to protect neurovascular units and strengthen spared functions of the spinal cord^119,120^. Moreover, exercise training can reduce the size of syringomyelia and the area of glial scar to promote the regeneration of axons, the remodeling of synapses, and the remyelination of axons^121^. In addition, by enhancing or replacing the residual spinal cord and muscle function, exercise training can remodel the neural circuit to promote the recovery of motor function^122^. Through remolding the function of skeletal muscle and cerebral cortex, exercise training can regulate the physiological metabolism function of spinal motor neurons, and improve the function from end effectors such as skeletal muscle to the cerebral cortex in different degrees^123^.

### Electrical stimulation

ES can accelerate axon growth and myelin sheath formation^124^, stimulate neurons to discharge bioelectric signal to strengthen muscle contraction, thus reconnecting the neural network of spinal cord^115^. Therefore, the baseline excitability of neural circuits is regulated by ES, such as epidural electrical stimulation (EES) and transcutaneous spinal cord stimulation (tSCS), leading to action potential within and between neural circuits by regulating excitability further or closer to the motor threshold^125^. ES also recruits local neurosuppressive neurotransmitters across synaptic sites by stimulating the amount of neurostimulation of afferent nerve fibers, thus enhancing the release capacity of neurotransmitters^126^. A clinical study has suggested that EES may promote the recovery of the sensorimotor network of the spinal cord after SCI by producing robust and coordinated spinal motor output, thus producing independent standing and gait^127^. In another clinical study, EES restored the voluntary control of walking in patients with severe or complete paralysis caused by SCI four years ago despite full rehabilitation^128^. TSCS is a new, non-invasive and promising technology, which can stimulate the spinal cord from the surface of the skin and reach the spinal cord network by using high current and unique waveform ES. For instance, Inanici and his colleagues used tSCS to significantly restore upper limb movement and sensation in an elderly patient with incomplete SCI, indicating that tSCS can promote neuroplasticity and long-term recovery^129^.

### Brain–computer interfaces

**Fig. 5:**
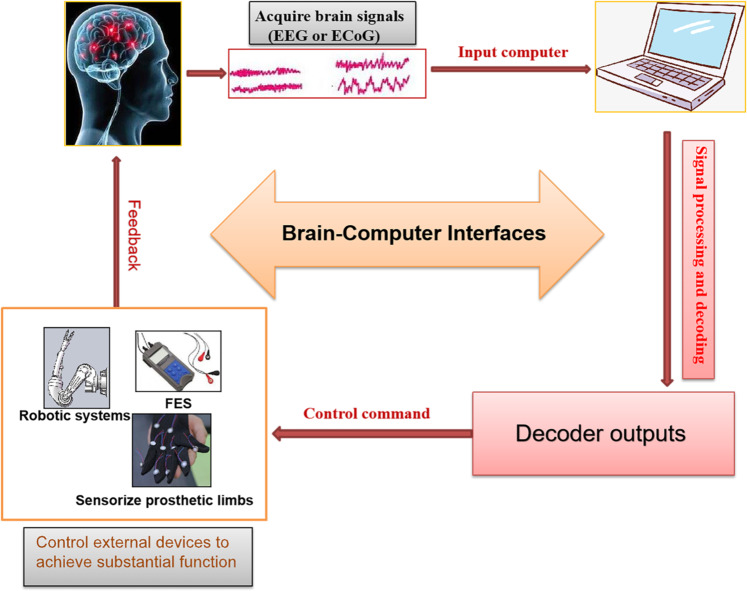
General diagram of BCIs for SCI. BCIs can control external devices to achieve substantial function by recording the electrical signals from the brain such as EEG or ECoG. The computer decodes the electronic signals from the motor cortex to drive robotic systems or stimulate muscles to recover the paralyzed person’s movement. Moreover, the combination of BCIs and sensory cortex can enhance the flexibility and fine control of limbs.

In recent years BCIs technology has become a research hotspot. BCIs, as the foundation of a new generation of the neuroprosthetic device, can control external devices to achieve substantial function by recording the electrical signals from the brain such as electroencephalography (EEG) or electrocorticography (ECoG)^130^ (Fig. 5). The prosthesis consists of implanted electrodes that record the expected motion of the paralyzed part of the body, computer algorithm that decode the expected motion, and auxiliary devices controlled by the expected motion signal^131^ (Fig. 5). The computer decodes the electronic signals from the motor cortex to drive robotic systems or stimulate muscles to recover the paralyzed person’s movement^132^. Moreover, the combination of BCIs and sensory cortex can enhance the flexibility and fine control of limbs^133^.

Combining BCIs with functional ES or robotic therapies provide hope for patients without residual motor function, which can retrain the connection between central and peripheral to reconstruct nerve circuits. BCIs combined with robotic systems such as exoskeletons and orthotics have become a new therapeutic modality to help or rehabilitate patients with limb disability. For example, Hochberg et al.^134^ reported that two patients with long-term quadriplegia used a BCI-based robotic arm to control and perform 3D stretching and grasping movements. The results showed that they could control the arm and hand over a wide space, and even one of them could use a robotic arm to drink coffee in the bottle. Benabid and his colleagues used an exoskeleton system controlled by an epidural wireless BCI to enable patients to use virtual avatars at home, control walking simulation procedures cortically, perform different stretching and touch tasks^135^. BCIs combined with functional electrical stimulation (BCIs-FES) can be used to activate the paralyzed muscle through ES^136^, transmit the electrical pulse directly to the peripheral nerve and muscle tissue to cause contraction and assist specific body movements^115^. For instance, a study conducted by Capogrosso et al. showed that the BCI-FES restored non-human primates weight-bearing movement of paralyzed legs on the treadmill and the ground six days after injury without prior training^137^. Another clinical study by Ajiboye et al. showed that the BCI-FES could successfully control coordinated movements of single-joint and multi-joint arms, achieve point-to-point target capture, and recover both stretching and grasping movements of patients with tetraplegia due to SCI^138^. Compared with motor function recovery, sensory function recovery is a higher requirement for SCI patients, and also a key step of BCI technology in clinical application. Microstimulation of the somatosensory cortex provides the potential to create sensory prosthetic limbs to restore sensory function^139^. For instance, a study conducted by O’Doherty et al. found that BCIs based-on sensory function recovery controlled the exploratory arrival movement of the actuator, and sent out the artificial tactile feedback signal via intracortical microstimulation (ICMS)^140^. ICMS of the primary somatosensory cortex can produce somatosensory sensation, which may become a method of sensory prosthesis in the future^141^.

### Combinatorial therapies

So far, due to the complexity of the pathological process of SCI, combinatorial therapies may be more effective, thus maximizing the effect of treatment of SCI. Some of these combinatorial strategies have been discussed in the previous paragraphs, and we will further develop them (Table 1). For example, MSCs improved the microenvironment of NSCs and promoted the survival of transplanted NSCs. Therefore, MSCs combined with NSCs could significantly improve the motor function after SCI^142^. Overexpression of IGF-1 in MSCs increased immune regulation, cell survival and myelination. Transplantation of IGF-1 overexpressed MSCs could significantly promote nerve regeneration after SCI^107^. IGF-1 inhibitd the inflammatory response and apoptosis via miR-219a-2-3p/YY1 mechanism and enhanced the neuroprotective effect of NSCs-derived exosomes, and their combinations significantly promoted the recovery of motor function^143^. The combination of gelatin sponge scaffold with NT-3 could create a favorable microenvironment, reduce inflammation and cavity formation, and induce the migration of endogenous nerve cells into the injury site to promote the regeneration of nerve and axon^144^. MnO_2_ NPs effectively enhanced the activity of MSCs by alleviating local oxidation environment, and hydrogel could promote cell adhesion growth and bridge spinal cord tissue. Therefore, combinations of MSCs and MnO2 NPs-dotted hydrogel induced neural differentiation of MSCs, resulting in the efficient regeneration of spinal cord tissue to significantly promote the recovery of motor function^145^. Co-culture of TrkC-gene-modified NSCs and NT-3-gene-modified SCs on the gelatin sponge scaffold to construct the neural network tissue. After transplanted into the SCI model in canine, it could significantly promote the regeneration of nerve fibers and integrate with the host neural circuit synapses, thus becoming a neuron relay to transmit the excitatory electrical signal through the lesion site^146^.

**Table 1:** Combinatorial strategies for SCI.

Combinatorial strategies	Animal model	SCI type	Follow up time	Comments about combination group	Reference
MSCs and NSCs	Rat	T10, contusion injury	8 weeks	MSCs improved the microenvironment of NSCs and promoted the survival of transplanted NSCs. MSCs combined with NSCs could significantly improve the motor function.	^142^
IGF-1 and MSCs	Mouse	T10, contusion injury	4 weeks	Overexpression of IGF-1 in MSCs increased immune regulation, cell survival and myelination. Transplantation of IGF-1 overexpressed MSCs could significantly promote nerve regeneration after SCI.	^107^
NSCs-derived exosomes and IGF-1	Rat	T10, contusion injury	4 weeks	IGF-1 inhibited the inflammatory response and apoptosis via miR-219a-2-3p/YY1 mechanism and enhanced the neuroprotective effect of NSCs-derived exosomes, and their combinations significantly promoted the recovery of motor function.	^143^
Gelatin sponge scaffold and NT-3	Rat and canine	T10, transection and hemisection	4 weeks	The combination of gelatin sponge scaffold with NT-3 could create a favorable microenvironment, reduce inflammation and cavity formation, and induce the migration of endogenous nerve cells into the injury site to promote the regeneration of nerve and axon.	^144^
MSCs and MnO2 NPs-dotted hydrogel	Rat	T10, transection	4 weeks	MnO2 NPs effectively enhanced the activity of MSCs by alleviating local oxidation environment, and hydrogel could promote cell adhesion growth and bridge spinal cord tissue. Therefore, combinations of MSCs and MnO2 NPs-dotted hydrogel induced neural differentiation of MSCs, resulting in the efficient regeneration of spinal cord tissue to significantly promote the recovery of motor function.	^145^
TrkC-gene modified NSCs, NT-3-gene modified SCs and gelatin sponge scaffold	Canine	T10, transection	24 weeks	Co-culture of TrkC-gene-modified NSCs and NT-3-gene-modified SCs on the gelatin sponge scaffold to construct the neural network tissue. it could significantly promote the regeneration of nerve fibers and integrate with the host neural circuit synapses, thus becoming a neuron relay to transmit the excitatory electrical signal through the lesion site.	^146^
MSCs and exercise training	Mouse	T10, aneurysm clip	8 weeks	Combinatorial treatment of MSCs and exercise training could promote the protection of nerve tissue and enhance the recovery of motor function after SCI.	^147^
EES and high-intensity treadmill training	Human	Not mentioned	85 weeks and 15 weeks	EES combined with high-intensity treadmill training enabled patients to walk on the ground, stand independently, and maintain trunk stability compared with individual exercise training.	^148^
BCIs, visuo-tactile feedback and assisted exercise training	Human	Not mentioned	112 weeks	Combinations of visual-tactile feedback, noninvasive BCIs and assisted exercise training significantly restored the patients’ motor function and part of sensory function, even part of intestinal, urinary and sexual functions.	^149^

However, the reconstruction of neural circuits after SCI also depends on rehabilitation strategies, such as exercise training, ES, and BCIs. For example, combinatorial treatment of MSCs and exercise training could promote the protection of nerve tissue and enhance the recovery of motor function after SCI^147^. EES combined with high-intensity treadmill training enabled patients to walk on the ground, stand independently, and maintain trunk stability compared with individual exercise training^148^. Combinations of visual-tactile feedback, noninvasive BCIs and assisted exercise training significantly restored the patients’ motor function and part of sensory function, even part of intestinal, urinary and sexual functions^149^.

## Conclusions and perspectives

The pathophysiology of SCI is complex and multifaceted, and its mechanisms and processes are incompletely understood. Thus, combinatorial therapies have been demonstrated to be more effective, and lead to better neural circuits reconstruction and functional recovery. Combinations of biomaterials, stem cells, growth factors, drugs and exosomes have been widely developed. However, simply achieving axon regeneration will not spontaneously lead to meaningful functional recovery. Therefore, the formation and remodeling of functional neural circuits also depend on rehabilitation exercises, such as exercise training, ES and BCIs.

It is believed that in the future we can analyze and record the nerve signals, and regulate the nerve signals through cell transplantation and molecular regulation after SCI. By stimulating local nerve circuits related to sensorimotor control and spinal cord autonomy, the protection, maintenance and even re-bridging of nerve circuits can be achieved. The relevant nerve circuits are fine-tuned, and then the corresponding nerve circuits are trained and strengthened by various rehabilitation methods. It can help the individual with SCI to resume autonomous movement as soon as possible, and ultimately promote the research and treatment of SCI to achieve more enormous breakthroughs in many fields.
